# Malignant Astroblastoma

**DOI:** 10.4274/balkanmedj.galenos.2020.2019.11.39

**Published:** 2020-06-01

**Authors:** Binnaz Sarper, Büşra Yaprak Bayrak, Hatice Halis, Burcu Alparslan, Hülya Yeni Bayraktar

**Affiliations:** 1 Department of Radiation Oncology, Kocaeli University School of Medicine, Kocaeli, Turkey; 2 Department of Pathology, Kocaeli University School of Medicine, Kocaeli, Turkey; 3 Clinic of Radiation Oncology, Sakarya University Training and Resaerch Hospital, Sakarya, Turkey; 4 Department of Radiology, Kocaeli University School of Medicine, Kocaeli, Turkey; 5 Clinic of Pathology, Ass. Prof. Dr. Mustafa Kemal Tavşanlı Government Hospital, Kütahya, Turkey

In February 2013, a 23-year-old man was admitted to the hospital with headache. Cranial magnetic resonance imaging (MRI) showed a homogeneous, well-demarcated, hyperintense mass on T2-weighted imaging and peritumoral hyperintensity due to edema. The mass showed no enhancement on T1-weighted imaging with contrast (Figure 1A). Surgical resection was recommended. However, the patient rejected surgery. In 2016, he presented again with severe headache. MRI showed hemorrhage and herniation (Figure 1B). The patient underwent emergency surgery, but the mass could not be completely excised.

Morphologically, this hypercellular neoplasm exhibited the following features. There were perivascular pseudorosettes with short, thickened cytoplasmic processes extending from cell bodies to vessel adventitia. In addition, there was vascular hyalinization with little fibrillar background, tumor necrosis with pseudopalisading, and high mitotic activity (Figure 1I). Immunohistochemically, glial fibrillary acidic protein (GFAP) was diffusely present in the epithelioid cells, mostly around the perivascular areas. The tumor cells showed diffuse positivity for S-100 and vimentin and focal positivity for epithelial membrane antigen (EMA). The Ki-67 proliferation index was calculated as 60% (Figure 1J). In light of these findings, the patient was diagnosed with a malignant astroblastoma.

On postoperative MRI, the residual tumor was evident behind the resection cavity (Figure 1C). Radiotherapy (RT) and concomitant temozolomide (TMZ) were started immediately after surgery. Six months after RT, a contrast-enhancing lesion was noticed, which was considered to be radiation necrosis (Figure 1D). A new, ring-enhancing focus was apparent 9 months after surgery (Figure 1E), and by 12 months, this focus had progressed (Figure 1F). Because of this evident lesion progression, TMZ was changed to bevacizumab, which resulted in decreased tumor enhancement (Figure 1G). However, new white matter intensities were detected. The patient’s general condition became progressively worse, 9 months after starting bevacizumab. The recurrent lesion enlarged rapidly and spread to the corpus callosum, basal ganglia, and cerebral hemisphere white matter bilaterally (Figure 1H). This later image was obtained just before the patient’s death in March 2018. Written informed consent for publication was obtained from the patient’s father.

Astroblastomas are recognized as a separate entity from astroglial tumors and have been classified as “other gliomas” (1). Histological diagnosis is made based on the presence of typical features, including astroblastic perivascular pseudorosette and hyalinization in a relatively well-defined mass. These tumors contain epithelioid cells, and vascular sclerosis is evident. Additional typical features include dot-like staining with EMA. The tumor usually stains positive with antibodies to GFAP, S-100 protein, and vimentin. Tumors, listed as “other neuroepithelial tumors” in the World Health Organization classification, are graded as either a low-grade or malignant variant. Malignant astroblastomas were described as having high cellularity in either a focal or multiple foci patterns, anaplasia, increased mitotic activity (>5 mitoses per high power fields), elevated proliferative index (>10%), microvascular proliferation, and necrosis.

It is difficult to differentiate astroblastoma from other glial tumors, such as ependymoma, angiocentric glioma, and glioblastomas. In ependymomas, the tumor cells have small nuclei and are generally more fibrillar with true rosettes while being less pleomorphic and sclerotic. Angiocentric gliomas are infiltrative neoplasms consisting of monomorphic bipolar spindle cells arranged in an angiocentric pattern. Although glioblastomas generally contain focal areas of perivascular pseudorosettes, differential diagnosis of high-grade astroblastoma with glioblastomas is difficult. Genetic studies can help distinguish between these two neoplasms.

Histologically, astroblastomas overlap with glioblastoma and especially with lower-grade gliomas. Studies have shown that even if histologically diagnosed as astroblastoma, the tumor often has molecularly heterogeneous properties and does not represent a single entity. Clinical, radiological, and histopathological correlations, and if necessary, genetic examination, are essential for accurate diagnosis (2).

Optimal treatment for astroblastomas is not yet clear. The gross total excision of the mass seems to be the most important variable that determines patient survival (3). In the literature, postoperative RT and chemotherapy have shown a variable, but gross total resection and RT have been the recommended treatment options in the case of malignant astroblastoma (4,5).

## Figures and Tables

**Figure 1:**
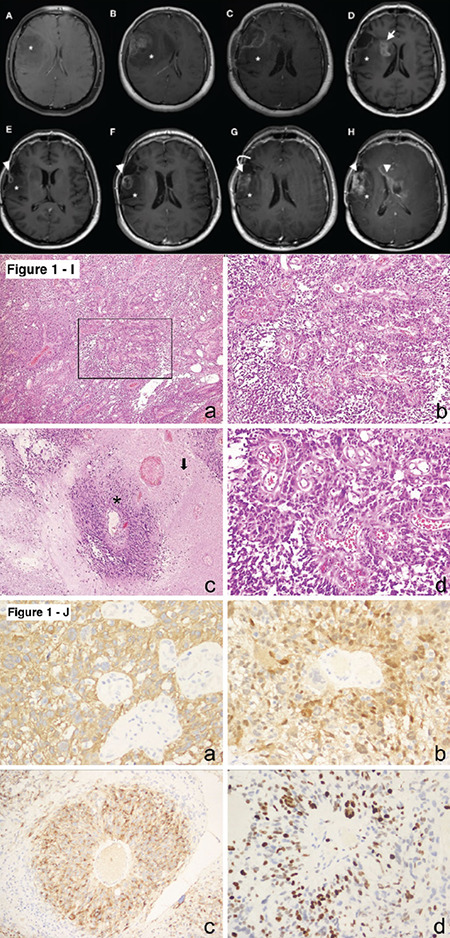
Postcontrast axial T1-weighted images: (A) Mass on first admission (*). (B) The preoperative tumor (*). (C) The postoperative residual tumor (*). (D) Sixth month after treatment: residual tumor (*) and radiation necrosis (√︎). (E) Ninth month after treatment: lesion (▼). (F) Twelfth month, progression. (G) Beginning of bevacizumab therapy, pseudoresponse (↳). (H) After bevacizumab therapy, rapid progression. Histopathological images: (Ia) Magnification ×40, glial neoplastic cells, and hematoxylin and eosin staining. (Ib) ×100 Magnified area of the continuous rectangular shape of image Ia. (Ic) ×100 Perivascular pseudorosette (*) and necrotic area (→). (Id) ×200 Magnified from image Ib. (J) Positive staining ×100 for (Ja) glial fibrillary acidic protein, (Jb) S-100, (Jc) epithelial membrane antigen, and (Jd) Ki67
